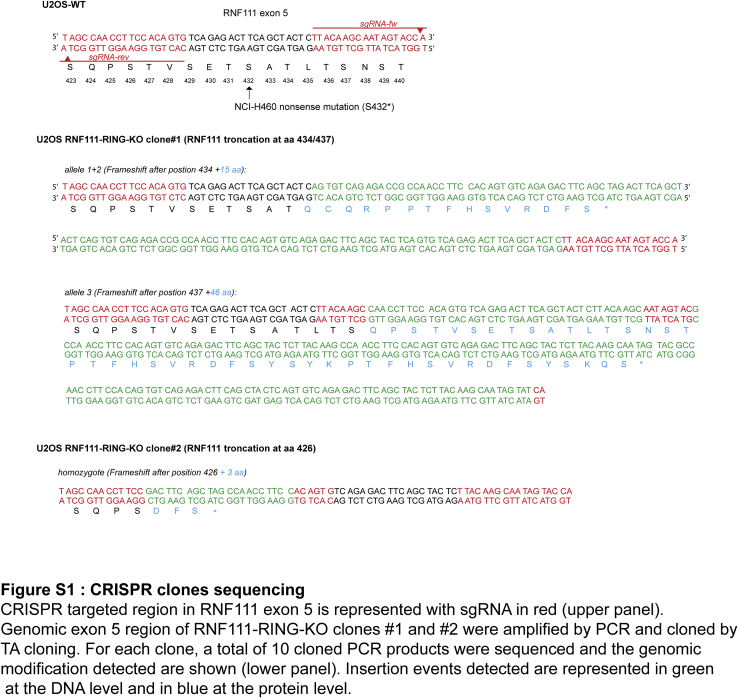# Quantitative Ubiquitylome Analysis Reveals the Specificity of RNF111/Arkadia E3 Ubiquitin Ligase for its Degradative Substrates SKI and SKIL/SnoN in TGF-β Signaling Pathway

**DOI:** 10.1016/j.mcpro.2021.100173

**Published:** 2021-11-03

**Authors:** Victor Laigle, Florent Dingli, Sadek Amhaz, Tiphaine Perron, Mouna Chouchène, Sabrina Colasse, Isabelle Petit, Patrick Poullet, Damarys Loew, Céline Prunier, Laurence Levy

**Affiliations:** 1 Institut Curie, PSL Research University, Laboratoire de Spectrométrie de Masse Protéomique, Paris, France; 2 Sorbonne Université, Inserm, Centre de Recherche Saint-Antoine, CRSA, Paris, France; 3 Institut Curie, PSL Research University, INSERM U900, Paris, France

**Keywords:** RNF111, SKIL, TGF-β, E3 ubiquitin ligase, proteomics, ubiquitylome, AGC, automatic gain control, diGly, diGlycine, FA, formic acid, FABP3, fatty acid binding protein 3, GDF15, growth differentiation factor 15, IAA, iodoacetamide, KYNU, Kynureninase, NCE, normalized collision energy, RNF111, ring finger protein 111, TGF-β, Transforming Growth Factor-β, UB, Ubiquitin, XIC, extracted ion chromatograms

## Abstract

RNF111/Arkadia is an E3 ubiquitin ligase that activates the transforming growth factor-β (TGF-β) pathway by degrading transcriptional repressors SKIL/SnoN and SKI. Truncations of the RING C-terminal domain of RNF111 that abolish its E3 function and subsequently activate TGF-β signaling are observed in some cancers. In the present study, we sought to perform a comprehensive analysis of RNF111 endogenous substrates upon TGF-β signaling activation using an integrative proteomic approach. In that aim, we carried out label-free quantitative proteomics after the enrichment of ubiquitylated proteins (ubiquitylome) in parental U2OS cell line compared with U2OS CRISPR engineered clones expressing a truncated form of RNF111 devoid of its C-terminal RING domain. We compared two methods of enrichment for ubiquitylated proteins before proteomics analysis by mass spectrometry, the diGlycine (diGly) remnant peptide immunoprecipitation with a K-ε-GG antibody, and a novel approach using protein immunoprecipitation with a ubiquitin pan nanobody that recognizes all ubiquitin chains and monoubiquitylation. Although we detected SKIL ubiquitylation among 108 potential RNF111 substrates with the diGly method, we found that the ubiquitin pan nanobody method also constitutes a powerful approach because it enabled the detection of 52 potential RNF111 substrates including SKI, SKIL, and RNF111. Integrative comparison of the RNF111-dependent proteome and ubiquitylomes enabled the identification of SKI and SKIL as the only targets ubiquitylated and degraded by RNF111 E3 ligase function in the presence of TGF-β. Our results indicate that lysine 343 localized in the SAND domain of SKIL constitutes a target for RNF111 ubiquitylation and demonstrate that RNF111 E3 ubiquitin ligase function specifically targets SKI and SKIL ubiquitylation and degradation upon TGF-β pathway activation.

## Highlights

• Identification of the endogenous RNF111 ubiquitylated substrates.
• A new powerful method to profile protein ubiquitylation using pan UB nanobody.
• Lysine K343 as a target for RNF111 ubiquitylation.
• SKI and SKIL are the only identified RNF111 degradative targets in TGF-β signaling.

## Graphical abstract

**Figure:**
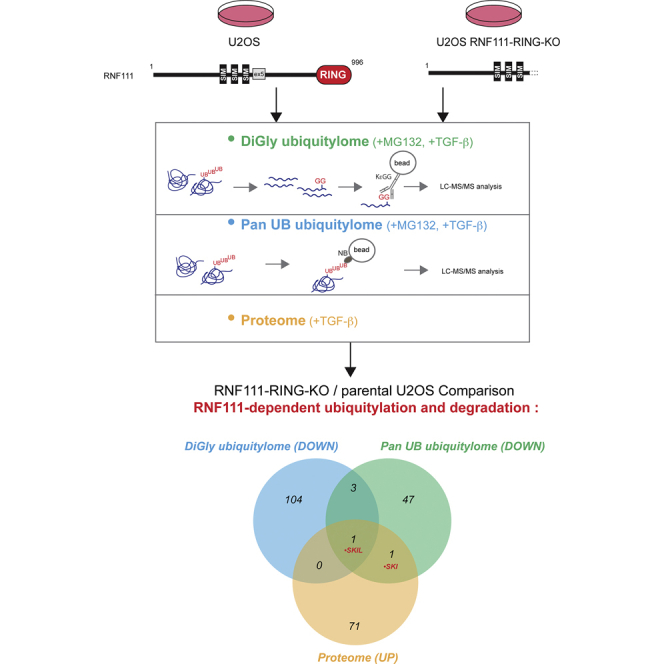

## In Brief

In this study, we aimed to identify exhaustively the substrates of the E3 ubiquitin ligase RNF111 that activates TGF-β signaling. We performed quantitative ubiquitylome comparison of parental U2OS cells with CRISPR modified clones that express a truncated RNF111 devoid of RING domain using two approaches based on the enrichment of ubiquitylated proteins. Integrative proteomics comparison of ubiquitylome and proteome identifies SKI and SKIL as the only targets ubiquitylated and degraded by RNF111 upon TGF-β stimulation.

The ubiquitin-proteasome system plays an important role in the regulation of many cellular signaling pathways by controlling protein stability. The ubiquitin-proteasome system involves ubiquitylation of proteins by E3 ubiquitin ligases that allows covalent attachment of the ubiquitin protein to a lysine residue on specific substrates, in cooperation with an E1-activating enzyme and an E2-conjugating enzyme. Polyubiquitylation can occur by polymerization of the ubiquitin molecules *via* one of its seven lysine residues (K6, K11, K27, K29, K33, K48, and K63) or its N-terminal methionine (M1), which generates as many different polyubiquitin linkages (1). This ubiquitylation code leads to distinct biochemical outcomes for the substrate, and it is admitted that only K48 polyubiquitylation, and to a lesser extent K11 polyubiquitylation, drive substrates toward degradation by the proteasome.

Transforming growth factor-β (TGF-β) pathway plays an important role in embryonic development and in tumor progression by inducing a large panel of target genes involved in cell cycle arrest, epithelial-mesenchymal transition, and cell migration mainly through activation of the SMAD2/3–SMAD4 transcription complex. The TGF-β signaling pathway is highly regulated by various E3 ubiquitin ligases such as SMURF1/2, TRIM33, WWP1, and RNF111 (also named Arkadia [ring finger protein 111]) (2, 3). We and others have found that RNF111 harbors a C-terminal RING domain required for its E3 ubiquitin ligase function that activates SMAD-dependent transcription in response to TGF-β by inducing degradation of SKI and SKIL (also named SnoN) transcriptional repressors (4, 5, 6). Although the RNF111-dependent SKI and SKIL degradation induced by TGF-β is clearly established, the mechanism for this inducible degradation is still puzzling, in particular the ubiquitylation events that underlie this degradation. RNF111 has also been reported to regulate the stability of SMAD7, an inhibitor of TGF-β signaling that acts at the TGF-β receptor level (7). Mutations that disrupt the C-terminal RING domain of RNF111 can occur in cancer (8), and we have shown that the NCI-H460 lung cancer cell line exhibits a S432∗ nonsense mutation that leads to the expression of a truncated form of RNF111 lacking its C-terminal RING domain. Such truncation abolishes SKI and SKIL degradation and subsequent SMAD-dependent transcription in response to TGF-β in this cancer cell line (9).

Although RNF111 has mainly been involved in the activation of TGF-β signaling, its E3 ubiquitin ligase function is not restricted to this pathway. RNF111 also contains 3 small ubiquitin-like modifier interacting motifs in its N-terminal region which confer to RNF111 small ubiquitin-like modifier-targeted ubiquitin ligase function involved in promyelocytic leukemia degradation in response to arsenic treatment (10) and in xeroderma pigmentosum ubiquitylation during DNA damage repair induced by UV (11). It has also been proposed that RNF111 is involved in Histone H4 neddylation during DNA damage repair induced by ionizing radiation (12) and in endocytosis by targeting the micro2 subunit of Clathrin adapter 2 complex (13). Hence, RNF111, like most E3 ligases, might target different substrates involved in different biological processes. However, all these substrates were characterized by protein interaction approaches, which are not the most relevant considering that E3 ubiquitin ligases interaction with their substrates tend to be labile and could lead to substrate degradation. Moreover, in most studies, ubiquitylation was detected by the overexpression of RNF111 and ubiquitin, which could lead to forced ubiquitylation. To prevent such biases, in the present study, we have sought to use an endogenous approach to comprehensively identify the substrates of RNF111. Because it represents a small proportion of a protein pool in the cell, the ubiquitylated proteins can be challenging to detect at the endogenous level. However, in the past years, different methods of enrichment for ubiquitylated proteins have been developed that allow profiling of ubiquitylated proteins by mass spectrometry (ubiquitylome) (14, 15, 16). The breakthrough came with the use of K-ε-GG antibody that immmunoprecipitates the di-glycin (diGly) remnant peptides obtained after trypsic digestion of the ubiquitin linked to its targeted lysine on a substrate (17, 18). Yet, this method requires a large amount of starting material because it only focuses on specific peptides at ubiquitylation sites and not the whole proteins. Thus, the development of alternative approaches is still needed to increase sensitivity and to simplify ubiquitylome analysis.

In order to profile the substrates of RNF111, we have generated U2OS osteosarcoma CRISPR modified cell lines that express a truncated form of RNF111 devoid of the C-terminal RING domain (RNF111-RING-KO) that mimics the truncation observed in the cancer cell line NCI-H460. To detect the degradative substrates of RNF111, we have performed label-free quantitative proteomics to compare the proteome of the RNF111-RING-KO clones with the parental U2OS upon TGF-β induction, which enabled the detection of SKI and SKIL among 73 candidates. Further analysis of selected candidates indicates that regulation of their protein level by RNF111 occurs at the transcriptional level. To identify more precisely the ubiquitylated substrates of RNF111, we performed label-free quantitative comparison of the ubiquitylome of RNF111-RING-KO clones to the parental U2OS upon TGF-β induction by two means. We used diGly enrichment with the K-ε-GG antibody and a new approach using a commercially available pan ubiquitin nanobody (referred to as pan UB in this study) that strongly interacts with monoubiquitylated and all linkage polyubiquitylated proteins. With the diGly antibody approach, among the 3641 proteins corresponding to the ubiquitylation sites quantified, we identified 108 proteins that are potentially ubiquitylated by RNF111, including SKIL on lysine 343; whereas the pan UB nanobody approach enabled the detection of 54 potential substrates including SKI, SKIL, and RNF111 among the 8547 proteins quantified, demonstrating that this new method is very robust for substrate identification of E3 ubiquitin ligases. Moreover, comparison of the two ubiquitylomes leads to detection of SKIL as the only validated common RNF111 substrate and integrative comparison of the ubiquitylomes and proteome identified SKI and SKIL as the only proteins both ubiquitylated and degraded by RNF111 upon TGF-β pathway activation, among the 7746 proteins quantified in the proteome analysis. Altogether, our findings indicate a strong specificity of RNF111 E3 ubiquitin ligase function for degradative ubiquitylation of SKI and SKIL in response to TGF-β.

## Experimental Procedures

### Cell Lines and Plasmids

U2OS human osteosarcoma, HEK-293 human embryonic kidney, and NCI-H460 human non-small cell lung carcinoma cell lines were cultured in Dulbecco's modified Eagle's medium (DMEM) (U2OS and HEK-293) or RPMI (NCI-H460) medium containing 10% fetal bovine serum, 100 U/ml penicillin and 100 μg/ml streptomycin at 37 °C in 5% CO_2_. The pMLM3636 expression vector for Streptococcus pyogenes Cas9 sgRNA was a gift from JK Joung laboratory (Addgene plasmid # 43860). The Cas9^D10A^ expression vector was a gift from G Church (Addgene plasmid #41816) (19), the CAGA_12_-Luc plasmid was described in (20), and the Renilla expression vector pRL-TK vector is from Promega. The pCMV10-3xFlag-RNF111-WT (Flag-RNF111-WT) expression vector was generated by PCR subcloning of human RNF111 cDNA (corresponding to isoform 3) from PcDNA4/TO-SFS-RNF111 (11) in PCMV10-3xFlag (Sigma). The pCMV-3xHA-SKIL-WT (HA-SKIL-WT) expression vector was generated by PCR subcloning of human SKIL cDNA form PCMV5B-HA-SnoN (21) in PCMV-3xHA. PCMV10-3xFlag-RNF111-C933A (Flag-RNF111-C933A) and pCMV-3xHA-SKIL-342/43-KR (HA-SKIL-342/43-KR) mutants were generated by site-directed mutagenesis respectively on pCMV10-3xFlag-RNF111-WT and pCMV-3xHA-SKIL-WT by using the QuickChange Lightning kit (Agilent).

### CRISPR Cell Lines

SgRNA-rev CACTGTGGAAGGTTGGCTAC and SgRNA-fw CTTACAAGCAATAGTACCAC targeting exon 5 of the human RNF111 gene were designed using the CRISPOR software (crispor.org) (22) to perform double-nicking. Double-stranded oligonucleotides with overhangs were cloned into BsmBI digested MLM3636 vector, and 0.5 million of U2OS cells were cotransfected with 2 μg of Cas9^D10A^ expression vector and 2 μg of each SgRNA-rev and SgRNA-fw MLM3636 expressing vector, using Amaxa Nucleofector V kit (Lonza), program X-001. The single cells were individually seeded in 96 well plates and the clones were amplified and assessed by Western-blot for full-length RNF111 depletion. RNF111 exon5 targeted region was PCR amplified from clones #1 and #2 genomic DNA with primers CATCTACCTCTGAGCAGGCC and TCATGCTTTTGGT GTCAGCC, and the PCR products were subcloned into pCR2.1 vector using TOPO-TA Cloning kit (Invitrogen). For each CRISPR clones, a total of ten cloned PCR products were sequenced to determine the genomic modification on the different alleles.

### Immunoprecipitation and Western Blot

Whole-cell extracts were prepared from 6-well plates and treated or not for 1 h with TGF-β (2 ng/ml) before lysis with RIPA buffer (50 mM Tris [pH 8], 150 mM NaCl, 1% NP-40, 0.5% sodium deoxycholate, and 0.1% sodium dodecyl sulfate) supplemented with EDTA-free protease Inhibitor (Roche), 50 μM NaF, and 50 μM β-glycerophospate. Cleared lysates were quantified by BCA protein assay (Pierce), and 30 μg of proteins were analyzed by Western blotting using standard procedures.

For pan UB nanobody immunoprecipitation on endogenous proteins, cells were grown to 90% confluence in 150 mm plates for each condition and treated with 10 μM MG132 for 4 h followed by 1 h TGF-β (2 ng/ml) treatment before lysis in RIPA buffer. The lysates were sonicated (10 s ON and 10 s OFF, four times) and the cleared lysates were quantified using BCA quantification. 6 mg of proteins were immunoprecipitated with 50 μl of Ubiquitin pan Selector beads slurry (Nanotag Biotechnologies, #N2510) on a rotator for 1 h at 4 °C. After three washes with RIPA buffer, the proteins were eluted in laemmli buffer before subsequent analysis by Western blotting using standard procedures.

For pan UB nanobody immunoprecipitation on transfected cells, cells grown in 6-well plates were transfected with 2 μg of the appropriate plasmids using X-tremGENE HP (Roche) and were treated 24 h later with 10 μM MG132 for 4 h followed by 1 h with 2 ng/ml TGF-β (U2OS) or 20 ng/ml activin A (HEK-293) before lysis in RIPA buffer. The sonicated and cleared lysates were immunoprecipitated with 20 μl of Ubiquitin pan Selector beads slurry (Nanotag Biotechnologies, #N2510) for 1 h and washed 3 times with RIPA buffer before analysis by Western blotting using standard procedures.

The following antibodies were used for Western blotting: anti-Flag-horseradish peroxidase (Sigma), anti-hemagglutinin (anti-HA-horseradish peroxidase; Roche), anti-RNF111 (M05, Abnova), anti-SKI (G8, Santa Cruz), anti-SKIL (19218-1-AP, Proteintech), anti-SMAD2/3 (BD), anti-UB (P4D1, Santa Cruz), anti-KYNU (E5, Santa Cruz), anti-GDF15 (G5, Santa Cruz), and anti-FABP3 (10676-1-AP, Proteintech).

### Luciferase Assay

For luciferase assay, cells grown in 24-well plates were cotransfected with 0.3 μg CAGA_12_-Luc and 0.2 μg pRL-TK (Promega). In the no TGF-β condition, 10 μM of TGF-β inhibitor (SB-431542, Torcis) was added at the time of transfection to inhibit autocrine TGF-β signaling in NCI-H460 cells. 24 h post transfection, TGF-β (2 ng/ml) was added or not for 8 h before lysis in passive lysis buffer (Promega), and successive measurements of Luciferase and Renilla activity with the dual-luciferase reporter assay system (Promega) were performed. The luciferase activities were normalized to Renilla activities in triplicate experiments.

### Quantitative RT-PCR

Total RNA was extracted with Trizol (Invitrogen) according to standard procedure from cells grown at 90% confluence in a 10 mm dish. cDNA were synthetized from 1.5 μg of RNA using the iScript cDNA synthesis kit (Bio-Rad). Quantitative PCR (qPCR) was performed in triplicate using the 2XSYBR Green qPCR master mix (Biotools) according to the manufacturer's protocol in a Light Cycler 96 (Roche). The expression of each gene was calculated by the 2^−ΔΔCt^ methods using GAPDH as a control. All data represent mean ± SD for at least three independent experiments. The following primers were used: GAPDH-F TGCACCACCAACTGCTTAGC, GAPDH-R GGCATG GACTGTGGTCATGAG, GDF15-F ACTCACGCCAGAAGTGCGG, GDF15-R AGATTCTGCCAGCAGTTGGTC, FABP3-F CTTCCCCCTA CCCTCAGGTG, FABP3-R CAGTGTCACAATGGACTTGACC, SKIL-F CAGCCTGATGCTCCGT GTAT, SKIL-R TGATGGTGCATCTGTCTTG GA, KYNU-F TTGCGGCT GAACTCAAATGC, and KYNU-R GCTTCCC CACTTCATGACCA.

### Ubiquitylome and Proteome Sample Preparation

For proteome analysis, the cells were grown to 90% confluence in a 6-well plates and treated with 2 ng/ml TGF-β for 1 h before lysis in freshly prepared urea buffer (8 M urea, 200 mM ammonium bicarbonate, and EDTA-free protease Inhibitor). After sonication, the lysates were quantified by BCA, and 300 μg of proteins were reduced with 5 mM DTT for 1 h at 37 °C and alkylated with 10 mM iodoacetamide (IAA) for 30 min at room temperature in the dark. The samples were then diluted in 200 mM ammonium bicarbonate to reach a final concentration of 1 M urea and digested overnight at 37 °C with trypsin (Worthington #LS003750) at a ratio of 1/50. 150 μg of each sample were separated with the High pH Reversed-Phase peptide fractionation kit (Pierce #84868). The peptides were eluted successively into six fractions using elution buffers containing the following percentages of acetonitrile: 10, 12.5, 15, 17.5, 20, and 50%. The eluted peptides were vacuum concentrated to dryness and resuspended in 20 μl of 0.1% formic acid (FA)/3% acetonitrile (vol/vol) before LC-MS/MS analysis.

For diGly ubiquitylome analysis, the samples were prepared according to the protocol described in (18). Briefly, for each condition, the cells were grown to 90% confluence in 8 x 150 mm plates and treated with 10 μM MG132 for 4 h after 1 h TGF-β (2 ng/ml) treatment before lysis in freshly prepared urea buffer (8M urea, 50 mM Tris-HCl (pH 7.5), 150 mM NaCl, 1 mM EDTA, 2 μg/ml aprotinin, 10 μg/ml leupeptin, 50 μM PR-619, 1 mM chloroacetamide, and 1 mM PMSF). The cleared lysates were quantified by BCA, and 10 mg of proteins were reduced and alkylated by adding successively 5 mM DTT and 10 mM IAA respectively for 1 h and 30 min at room temperature. The protein samples were diluted 4 times with 50 mM Tris-HCL to obtain a concentration at 2 M urea. Trypsin (Worthington # LS003750) digestion was then performed overnight at 37 °C at a ratio of 1/50. After centrifugation at 3000*g* for 5 min, supernatant containing the digested peptides were desalted on a 500-mg tC18 SepPak cartridge, and the eluted peptides were dried by vacuum centrifugation. The peptides were resuspended in 1.4 ml IAP buffer (50 mM MOPS (pH 7.2), 10 mM sodium phosphate Na2HPO_4_, and 50 mM NaCl), cleared at 20,000*g* for 5 min, and incubated on a rotator for 2 h at 4 °C with 50 μl of PTMScan Ubiquitin Remnant Motif (K-ε-GG) Antibody Beads slurry (Cell Signaling #5562) equilibrated in IAP buffer. After two washes in IAP buffer followed by three washes with milliQ water, two successive elutions of the K-ε-GG peptides with 55 μl of TFA 0.15% for 10 min were combined and desalted using C18 StageTips. The final peptide eluates were dried and resuspended in 8 μl 0.1% FA/3% (vol/vol) acetonitrile before LC-MS/MS analysis.

For pan UB nanobody Ubiquitylome, immunoprecipitation on the endogenous proteins was performed as described in the immunoprecipitation section, except that the three washes with RIPA buffer were followed by 2 washes with washing buffer (150 mM NaCL and 50 mM TRIS pH 7.5), and the proteins were subsequently eluted twice with 150 μl of freshly prepared solution of 1.4% triethylamide for 5 min at room temperature under agitation. The 300 μl triethylamide eluates were neutralized with 100 μl of Tris 1 M pH 7.5 and dried by vacuum centrifugation. The proteins were reduced and alkylated by adding successively 5 mM DTT and 10 mM IAA, as previously described. The samples were then diluted in 400 μl of 25 mM ammonium bicarbonate, digested at 37 °C for 2 h with 0.4 μg of trypsin/LysC (#V5073 Promega) before overnight digestion by adding 1 μg of trypsin/LysC. The sample were then loaded onto homemade SepPak C18 Tips packed by stacking one AttractSPE disk (#SPE-Disks-Bio-C18–100.47.20, Affinisep) and 2 mg beads (SepPak C18 #186004521, Cartridge Waters) into a 200 μl micropipette tip for desalting. The peptides were eluted using 40/60 acetonitrile/H2O in 0.1% FA and vacuum concentrated to dryness. The sample was resuspended in 10 μl of TFA 0.3% before LC-MS/MS analysis

### Ubiquitylome and Proteome Analysis by LC-MS/MS

Peptides for proteome analyses were separated by reversed phase LC on an RSLCnano system (Ultimate 3000, Thermo Scientific) coupled online to an Orbitrap Fusion Tribrid mass spectrometer (Thermo Scientific). The peptides were trapped on a C18 column (75 μm inner diameter × 2 cm; nanoViper Acclaim PepMap 100, Thermo Scientific) with buffer A (2/98 acetonitrile/H_2_O (vol/vol) in 0.1% FA) at a flow rate of 4.0 μl/min over 4 min. The separation was performed on a 50 cm x 75 μm C18 column (nanoViper Acclaim PepMap RSLC, 2 μm, 100 Å, Thermo Scientific) regulated to a temperature of 55 °C with a linear gradient of 5 to 25 % buffer B (100% acetonitrile, 0.1% FA) at a flow rate of 300 nl/min over 100 min. The peptides were ionized by a nanospray ionization ion source at 2.2 kV. Full-scan MS in the Orbitrap was set at a scan range of 400 to 1500 with a resolution at 120,000 (at 200 m/z), and the ions from each full scan were fragmented in higher-energy collisional dissociation mode and analyzed in the linear ion trap in rapid mode. The fragmentation was set in top speed mode in data-dependent analysis. We selected ions with charge state from 2+ to 6+ for screening. Normalized collision energy (NCE) was set to 30, automatic gain control (AGC) target to 20,000 ions with a dynamic exclusion of 30s.

For diGly ubiquitylome analyses, LC was performed as previously described with an RSLCnano system (same trap column, column, and buffers), coupled online to a Q Exactive HF-X mass spectrometer (Thermo Scientific). The peptides were trapped onto the C18 column with buffer A at a flow rate of 2.5 μl/min over 4 min. Separation was performed at a temperature of 50 °C with a linear gradient of 2 to 30% buffer B at a flow rate of 300 nl/min over 91 min. The peptides were ionized by a nanospray ionization ion source (voltage was 2.2 kV). MS full scans were performed in the ultrahigh-field Orbitrap mass analyzer in ranges m/z 375 to 1500 with a resolution of 120,000 (at 200 m/z) and detected in the Orbitrap analyzer after accumulation of ion at 3E6 target value with a maximum injection time (IT) of 50 ms. For every full scan, the top 20 most intense ions were isolated (isolation width of 1.6 m/z) and fragmented (NCE of 27) by higher-energy collisional dissociation mode with an IT of 60 ms, AGC target set to 1E5, and 15,000 resolution. The charge state from <2+ and >6+ were excluded, and dynamic exclusion was set to 40s.

For pan UB ubiquitylome analyses, LC was performed as previously described with an RSLCnano system, coupled online to an Orbitrap Exploris 480 mass spectrometer (Thermo Scientific). The peptides were trapped on a C18 column with buffer A at a flow rate of 3.0 μl/min over 4 min. Separation was performed at a temperature of 40 °C with a linear gradient of 3% to 32% buffer B at a flow rate of 300 nl/min over 211 min. MS full scans were performed in the ultrahigh-field Orbitrap mass analyzer in ranges m/z 375 to 1500 with a resolution of 120,000 (at m/z 200), AGC target value set at 300 % and with a maximum IT of 25 ms. The top 30 most intense ions were isolated (isolation width of 1.6 m/z) and fragmented with a NCE set at 30%, a resolution of 15,000, and AGC target value set to 100%. We selected ions with charge state from 2+ to 6+ for screening and dynamic exclusion of 40s.

#### Mass Spectrometry Data Analysis

For identification, the raw MS files were searched against the *Homo sapiens* UniProt database (UP000005640, downloaded 11/2017 with 20,239 entries for the diGly ubiquitylome, 01/2018 with 20,231 entries for the proteome, and 12/2019 with 20,364 entries for the pan UB ubiquitylome), combined with common contaminants (245 sequences, downloaded from http://www.coxdocs.org/doku.php?id=maxquant:start_downloads.htm the 27/07/2016) for the diGly ubiquitylome analyses. The proteome and pan UB ubiquitylome samples being sufficiently complex, the non human contaminants were negligible and were not added to the search. The search was conducted using SEQUEST-HT through Proteome Discoverer (version 2.1 for the diGly ubiquitylome, 2.2 for the proteome, and 2.4 for the pan UB ubiquitylome) after the Spectrum Selector node with default settings. Enzyme specificity was set to trypsin (full), and a maximum of two miscleavage sites were allowed for the proteome and pan UB ubiquitylome and three for the diGly ubiquitylome. Oxidized methionine, Carbamidomethyl cysteines, and N-terminal acetylation were set as variable modifications, and GlyGly on lysine (+114.0429) was added for the ubiquitylomes analyses. Methionine-loss and Methionine-loss + N-terminal acetylation were also added to the pan UB variable modifications. For all analyses, the maximum allowed mass deviation was set to 10 ppm for monoisotopic precursor ions. For fragment ions, it was set respectively to 0.6 Da and 0.02 Da for the proteome and the ubiquitylomes. For proteome analyses, the Top N peaks filter of Proteome Discoverer was set to the six most intense peaks every 100 Da. FDR calculation used Percolator (23) and was set to the conventional threshold of 1% at the peptide level for the whole study. The resulting files were further processed using myProMS v3.9 (24) (code available at https://github.com/bioinfo-pf-curie/myproms). The label-free quantification was performed using peptide Extracted Ion Chromatograms (XICs) computed with MassChroQ (25), version 2.2.21. For proteome and ubiquitylome quantifications, XICs from all proteotypic peptides shared between compared conditions (TopN matching) were used, and the missed cleavages were allowed. All peptides data, including quantification, are available from ProteomeXchange with identifier PXD025890, and the proteins and ubiquitylation sites data (identification and quantification) are available in supplemental Tables S1–S4.

#### Experimental Design and Statistical Rationale

Proteome analyses were performed on three biological replicates for the parental U2OS cells, treated with TGF-β and used as controls (n = 3), and three for each of RNF111-RING-KO clones #1 and #2 (n = 6 biological replicates in total for RNF111-RING-KO), to accommodate biological variability. Each replicate was divided into six fractions for MS analysis, and XICs were summed across the fractions for each peptide before statistical analysis. DiGly ubiquitylome analyses were performed on the parental U2OS cells and on the RNF111-RING-KO clone #2, with three biological replicates each. Each sample was analyzed twice (2 technological replicates), and the obtained XICs were merged (averaged when measured twice) to improve the number of ubiquitylation sites identified and quantified. For the pan UB ubiquitylome analysis, four biological replicates for parental U2OS cell line (n = 4) were compared with four biological replicates for each of RNF111-RING-KO clones #1 and #2 (n = 8 for the RNF111-RING-KO condition).

For each experiment, statistical analysis was then performed inside myProMS v3.9 (24), after checking for normal distribution. The identified contaminants were excluded from the analysis at this point for the diGly ubiquitylome samples. Median and scale normalization was applied on the total signal to correct the XICs for each biological replicate. Outlier peptides were removed, within each condition and for each protein, with the Tukey's fences method. To estimate the significance of the change in protein abundance, a linear model (adjusted on peptides and biological replicates) was used, and *p*-values were adjusted with the Benjamini–Hochberg FDR procedure. In addition, proteins/sites specific to a single condition were also included in the analysis. Candidates for RNF111 ubiquitylation substrates or sites were retained under the criteria of a minimum 2-fold change between RNF111-RING-KO and the parental U2OS cells (increase in proteome, decrease in diGly and pan UB ubiquitylomes), which must be statistically significant as reported by an adjusted *p*-value under 0.05, and the candidates must have at least one corresponding peptide identified in three replicates of any of the two conditions.

## Results

### CRISPR Engineered U2OS RNF111-RING-KO Cell Lines are not Responsive to TGF-β

**Fig. 1:**
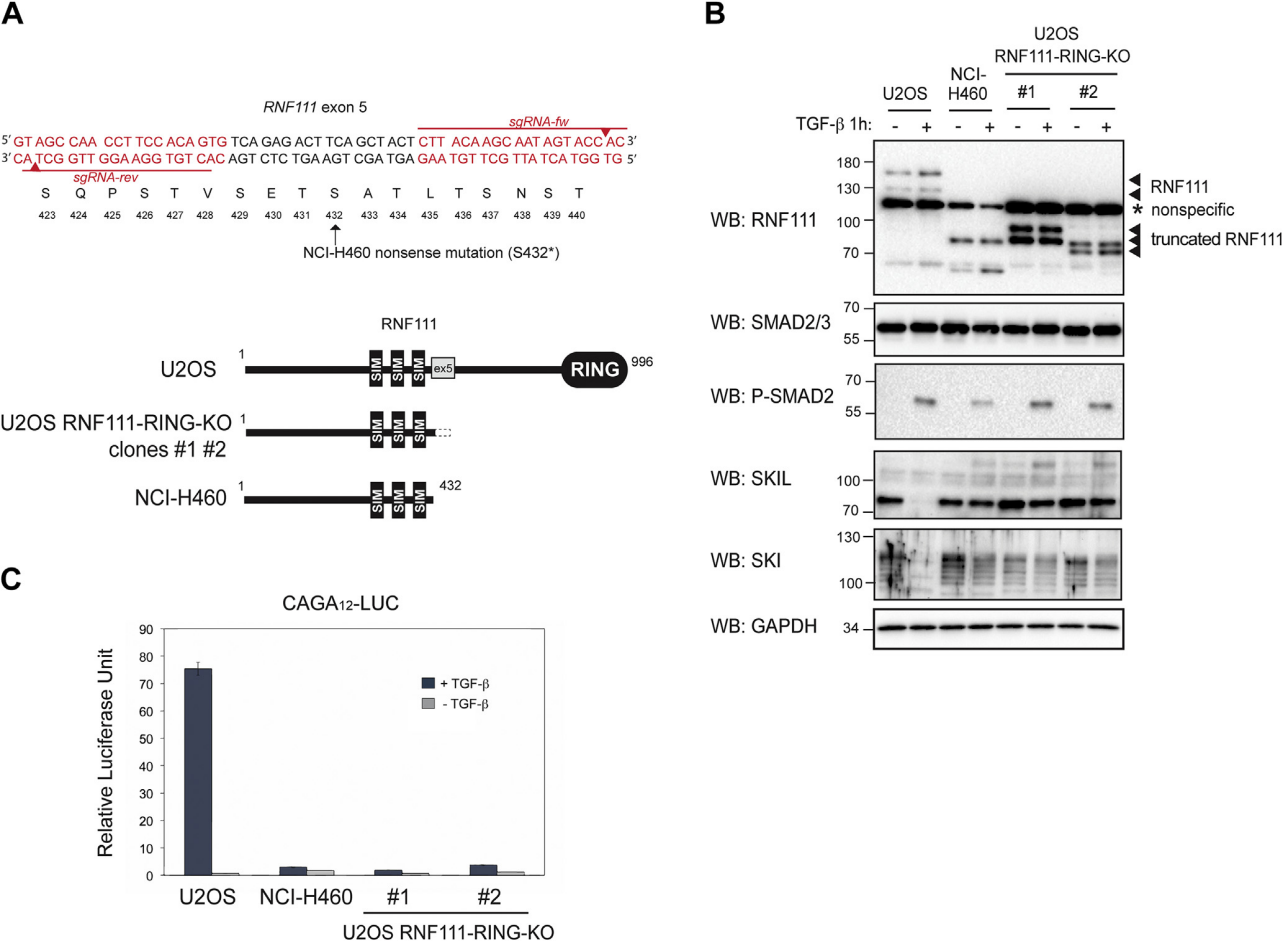
**CRISPR engineered U2OS RNF111-RING-KO cell lines.***A*, *upper diagram*, design of the reverse and forward sgRNA (sgRNA-rev and sgRNA-fw, in *red*) used to target exon 5 of RNF111 gene in U2OS cells. *Red arrows* indicate the breaking sites. Amino acids of the corresponding codons are annotated below; nonsense mutation on serine 432 observed in NCI-H460 cell line is indicated (S432∗). *Lower diagram*, schematic representation of WT RNF111 in parental U2OS cell line compared with RNF111 truncation in U2OS CRISPR engineered RNF111-RING-KO clones #1 and #2 and NCI-H460 cell line. *B*, U2OS cells, NCI-H460 cells, and RNF111-RING-KO clones #1 and #2 were treated or not with TGF-β for 1 h. The whole cell extracts were analyzed by Western blotting using antibodies against RNF111, SMAD2/3, P-SMAD2, SKIL, SKI, and GAPDH. *C*, U2OS cells, NCI-H460 cells, and RNF111-RING-KO clones #1 and #2 were cotransfected with the CAGA_12_-Luc and pRL-TK reporters and were treated or not with TGF-β for 8 h. The data represent means ± SD of luciferase activities normalized to Renilla in triplicate experiments. RNF111, ring finger protein 111; TGF-β, transforming growth factor-β.

To identify endogenous substrates of RNF111 E3 ubiquitin ligase function, we generated CRISPR engineered cell lines devoid of RNF111 RING domain by mimicking the stop mutation S432∗ observed on exon 5 of the RNF111 gene in the NCI-H460 carcinoma cell line. Considering the role of RNF111 in both TGF-β signaling and DNA repair, we set our study in the U2OS osteosarcoma cell line that exhibits an intact functional response to both TGF-β and DNA damage. We used the CRISPR/Cas9^D10A^ double nicking system that reduces off-target cleavage by 50 to 1500-fold in cell lines (26) to generate highly specific double strand break with paired single guide RNA (sgRNA-rev and sgRNA-fw) located on either side of the targeted region in exon 5 of RNF111 in U2OS cell line (Fig. 1*A*). The CRISPR single clones were selected by Western blotting with an antibody that recognizes the N-terminal region (amino acids 1–108) of RNF111 (Abnova M05). Two independent U2OS clones that express truncated forms of RNF111 with size range equivalent to the one observed in NCI-H460 cells, and no full length RNF111, were selected (referred to as RNF111-RING-KO clones #1 and #2) (Fig. 1*B*). Further sequencing of the genomic region of exon 5 indicates that RNF111-RING-KO clone #1 carries two alleles with a frameshift mutation at position 434 followed by 15 extra amino acids before stop mutation and 1 allele with a frameshift mutation at position 437 followed by 46 extra amino acids; whereas RNF111-RING-KO clone #2 carries the same frameshift mutation at position 426 followed by three extra amino acids on all alleles (supplemental Fig. S1).

As observed in NCI-H460 cell line (9), RNF111-RING-KO clones #1 and #2 undergo an intact SMAD2 phosphorylation upon TGF-β signaling after 1 h treatment with TGF-β, but the degradation of the SMAD transcriptional repressors SKI and SKIL is abolished in these two clones as compared with the parental U2OS cell line (Fig. 1*B*). As a consequence, the SMAD-dependent transcription in response to TGF-β assessed by luciferase assay with the CAGA_12_-LUC reporter is completely abolished in the two RNF111-RING-KO clones, as observed in the NCI-H460 cell line (Fig. 1*C*). Altogether, our results confirm that the two independent CRISPR engineered U2OS RNF111-RING-KO clones #1 and #2 have lost the E3 ubiquitin ligase function responsible for SKI and SKIL degradation upon TGF-β and are subsequently not responsive to TGF-β.

### Identification of the RNF111-Dependent Proteome

**Fig. 2:**
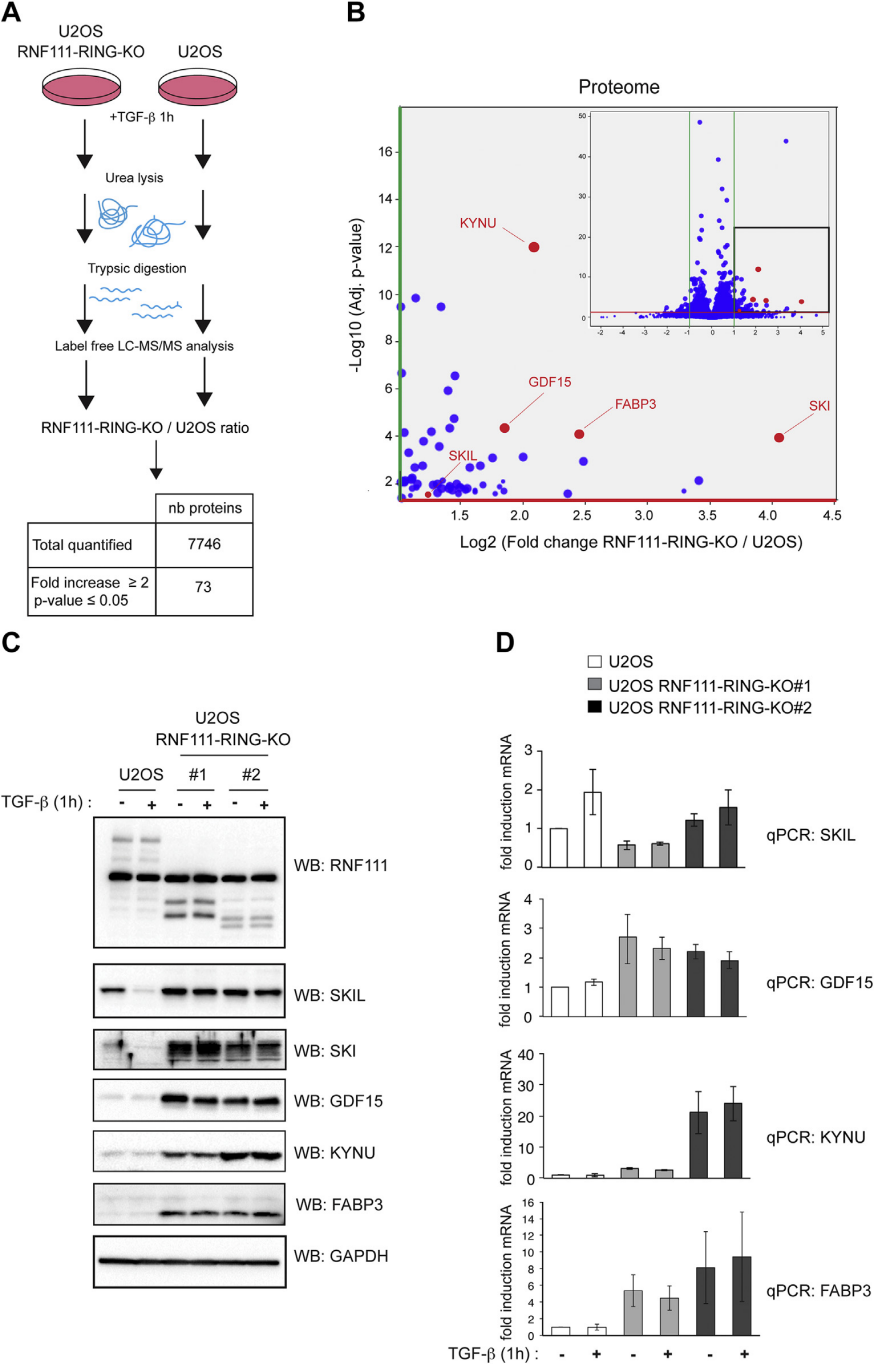
**RNF111-dependent Proteome.***A*, schematic representation of the proteome experiment with a summary table of the RNF111-RING-KO/U2OS protein ratio quantification results. *B*, volcano plot representation of the differential analysis with the log_2_ fold change RNF111-RING-KO/U2OS *versus* the negative log_10_*p*-value. Each dot represents a protein. *Green* and *red lines* represent the cut-off applied (respectively fold increase or decrease ≥2 and *p*-value ≤ 0.05). Volcano Plot is zoomed in the fold increase ≥ 2 and *p*-value ≤ 0.05 areas. *C*, Western blot analysis of the proteome candidates. U2OS cells and RNF111-RING-KO clones #1 and #2 were treated or not with TGF-β for 1 h. The whole cell extracts were analyzed by Western blotting using antibodies against RNF111, SKIL, SKI, GDF15 (growth differentiation factor 15), KYNU, FABP3 (fatty acid binding protein 3), and GAPDH. *D*, qPCR analysis of the proteome candidates. U2OS cells and RNF111-RING-KO clones #1 and #2 were treated or not with TGF-β for 1 h. The levels of mRNA for SKIL, GDF15, KYNU, and FABP3 were analyzed by qPCR and normalized to GAPDH using the 2^−ΔΔCt^ methods. The data represent mean ± SD for at least three independent experiments. KYNU, Kynureninase; RNF111, ring finger protein 111; TGF-β, transforming growth factor-β.

To identify proteins that are degraded by RNF111 E3 ligase function, we performed label-free quantitative proteomics on U2OS parental cells and RNF111-RING-KO clones #1 and #2 (Fig. 2*A*). We compared three biological replicates for each RNF111-RING-KO clones #1 and #2 (RNF111-RING-KO clones, n = 6) to three biological replicates for U2OS parental cells (U2OS, n = 3) to quantify RNF111-RING-KO/U2OS protein ratios. To detect RNF111-induced protein degradation that depends on active TGF-β pathway, as for SKI and SKIL degradation, the cells were treated with TGF-β for 1 h before lysis. This timing of TGF-β induction allows Phospho-SMAD2 accumulation and SKI and SKIL degradation but is too early to enable detection of the TGF-β target genes induction at the protein level. Significant increase of protein quantity in RNF111-RING-KO clones compared with parental U2OS cells (RNF111-RING-KO/U2OS fold increase ≥ 2; *p*-value ≤ 0.05) was detected for 73 proteins including SKI and SKIL among the 7746 proteins quantified (Fig. 2, *A* and *B*, supplemental Table S1). We further validated by Western-blot that Kynureninase (KYNU), growth differenciation factor 15 (GDF15), and fatty acid binding protein 3 (FABP3), some of the strongest candidates with commercially available antibodies, are indeed increased in both RNF111-RING-KO clones #1 and #2 as compared with parental U2OS cells, but unlike SKI and SKIL, we found that this increase is independent of TGF-β (Fig. 2*C*). Q-PCR analysis of these candidates indicate that their RNA levels are also increased in RNF111-RING-KO clones #1 and #2 compared to parental U2OS cells independently of TGF-β, whereas, as expected, SKIL RNA level is not (Fig. 2*D*). These results suggest that RNF111 downregulates KYNU, GDF15, and FABP3 directly or indirectly at the transcriptional level, rather than affecting their protein stability as for SKI and SKIL.

### Identification of RNF111-Dependent Substrates by Label-Free Quantitative Comparison of diGly Ubiquitylome

**Fig. 3:**
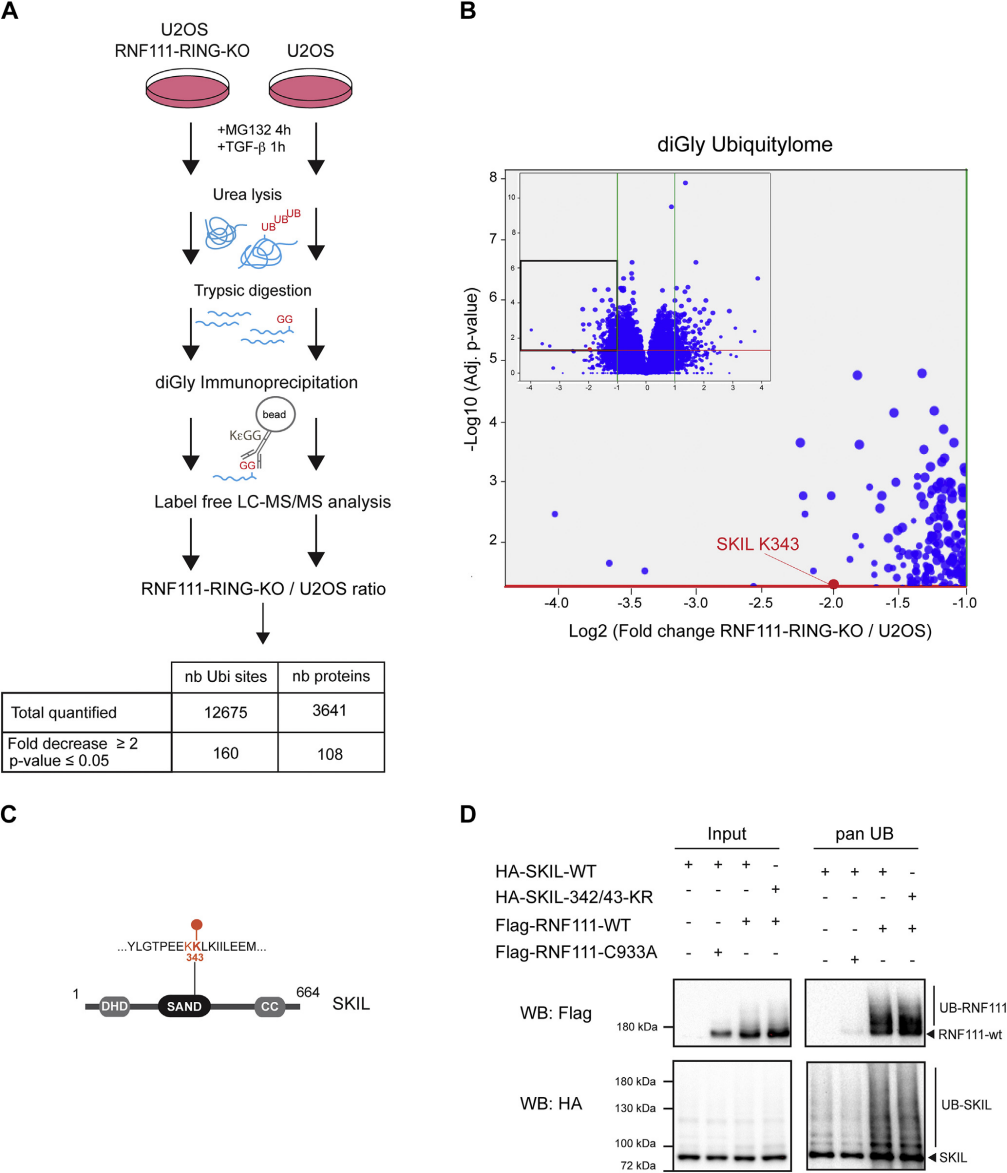
**Identification of RNF111-dependent substrates by diGly ubiquitylome quantitative comparison.***A*, schematic representation of the experimental design for RNF111-dependent ubiquitylome quantification with the diGly approach using immunoprecipitation of ubiquitylated trypsic remnant peptides with the K-ε-GG antibody. The table summarizes the results for the ubiquitylation sites quantification of the RNF111–RING–KO/U2OS ratio. *B*, volcano plot representation of the differential analysis with the log_2_ fold change RNF111-RING-KO/U2OS *versus* the negative log_10_*p*-value. Each dot represents a ubiquitylation site. *Green* and *red lines* represent the cut-off applied (respectively fold increase or decrease ≥2 and *p*-value ≤ 0.05). Volcano Plot is zoomed in the fold decrease ≥ 2 and *p*-value ≤ 0.05 area. *C*, schematic representation of the SKIL protein with its domains DHD, SAND, and CC (coild-coild) and the localization of lysine 343. Lysines 342 and 343 that were subsequently mutated are indicated in *red*. *D*, HEK-293 cells were transfected with Flag-RNF111-WT or Flag-RNF111-C933A catalytic inactive mutant along with HA-SKIL-WT, HA-SKIL-342/43-KR mutant, or empty vector. After 1 h Activin/TGF-β treatment, the whole cell extracts were immunoprecipitated with the pan UB nanobody and analyzed by Western blotting (pan UB, *right panel*) along with the corresponding whole cell extract (input, *left panel*) using HA and Flag antibodies. diGly, diglycine; RNF111, ring finger protein 111; UB, ubiquitin.

Because the RNF111-dependent proteome is crowded with proteins that are modified at the transcriptional level and considering that RNF111 might also perform non degradative ubiquitylation (11, 27), we went on to investigate directly global endogenous substrates ubiquitylated by the RING domain of RNF111. In that aim, we first performed diGly peptides profiling. The lysates were digested with trypsin, and the peptides carrying diGly modified lysine remnant of ubiquitylation were immunoprecipitated with the K-ε-GG antibody (Cell Signaling) (Fig. 3*A*). Three biological replicate experiments were performed to compare global ubiquitylation in parental U2OS cells (n = 3) and RNF111-RING-KO clone #2 (n = 3). To identify RNF111 substrates that are dependent of an active TGF-β signaling, we treated the cells with TGF-β for 1 h before lysis, which corresponds to the peak of SKIL degradation before detection of TGF-β target genes induction at the protein level. To prevent the proteasomal degradation of ubiquitylated substrates, the cells were treated with MG132 for 4 h before TGF-β induction. We assumed that these experimental conditions enable the detection of both TGF-β dependent and independent ubiquitylation events, as well as degradative and non degradative ubiquitylation.

Label-free quantification of ubiquitylation sites by mass spectrometry enabled the identification of 160 sites within 108 proteins, among 12,675 sites quantified within 3641 proteins, that display a significant decrease in RNF111-RING-KO clone #2 compared to the U2OS parental cells (RNF111-RING-KO/U2OS fold decrease ≥ 2; *p*-value ≤ 0.05; Fig. 3, *A* and *B*; supplemental Tables S2 and S3).

Among the 160 sites of ubiquitylation that significantly decrease in RNF111-RING-KO clone #2, we identified lysine 343 (K343) as an RNF111-dependent ubiquitylation target for SKIL (Fig. 3*B*). Interestingly, this lysine is localized in the SAND domain of SKIL (aa 258–353) that interacts with SMAD4 (28) and RNF111 (5) (Fig. 3*C*). Of note, one other ubiquitylation site was detected for SKIL on lysine 432 that does not display any significant changes between RNF111-RING-KO clone #2 and U2OS cells (supplemental Table S3). To determine if lysine 343 of SKIL is the lysine responsible for the degradation of SKIL induced by RNF111 in response to TGF-β signaling, we have mutated lysine 343 on a HA-SKIL expression vector. Lysine 343 is adjacent to lysine 342, it is therefore likely that lysine 342 could be ubiquitylated by RNF111 in the absence of lysine 343. To overcome this possibility, we performed lysine to arginine mutation of the two lysines 342 and 343 to generate a HA-SKIL-342/43-KR mutant. We have compared the ability of Flag-RNF111-WT or its inactive RING mutant Flag-RNF111-C933A to ubiquitylate HA-SKIL-WT or the HA-SKIL-342/43-KR mutant in the presence of TGF-β/Activin signal in HEK-293 cells. To immunoprecipitate ubiquitylated proteins, we used the newly developed pan UB nanobody resin (Ubiquitin pan Selector, Nanotag Biotechnologies) that exhibits strong affinity for polyubiquitylated and monoubiquitylated proteins. As expected, because RNF111 auto-ubiquitylates, Flag-RNF111-WT was immunoprecipitated, but not its catalytic inactive mutant C933A (Fig. 3*D*). Moreover, RNF111-WT, but not the C933A mutant, induces ubiquitylation of HA-SKIL-WT. However, we could not detect any decrease in SKIL ubiquitylation when lysines 342/43 were mutated, indicating that RNF111 might also ubiquitylate other lysines on SKIL that were not detected in the diGly ubiquitylome.

### Identification of RNF111-Dependent Substrates by Label-Free Quantitative Comparison of Pan UB Ubiquitylome

**Fig. 4:**
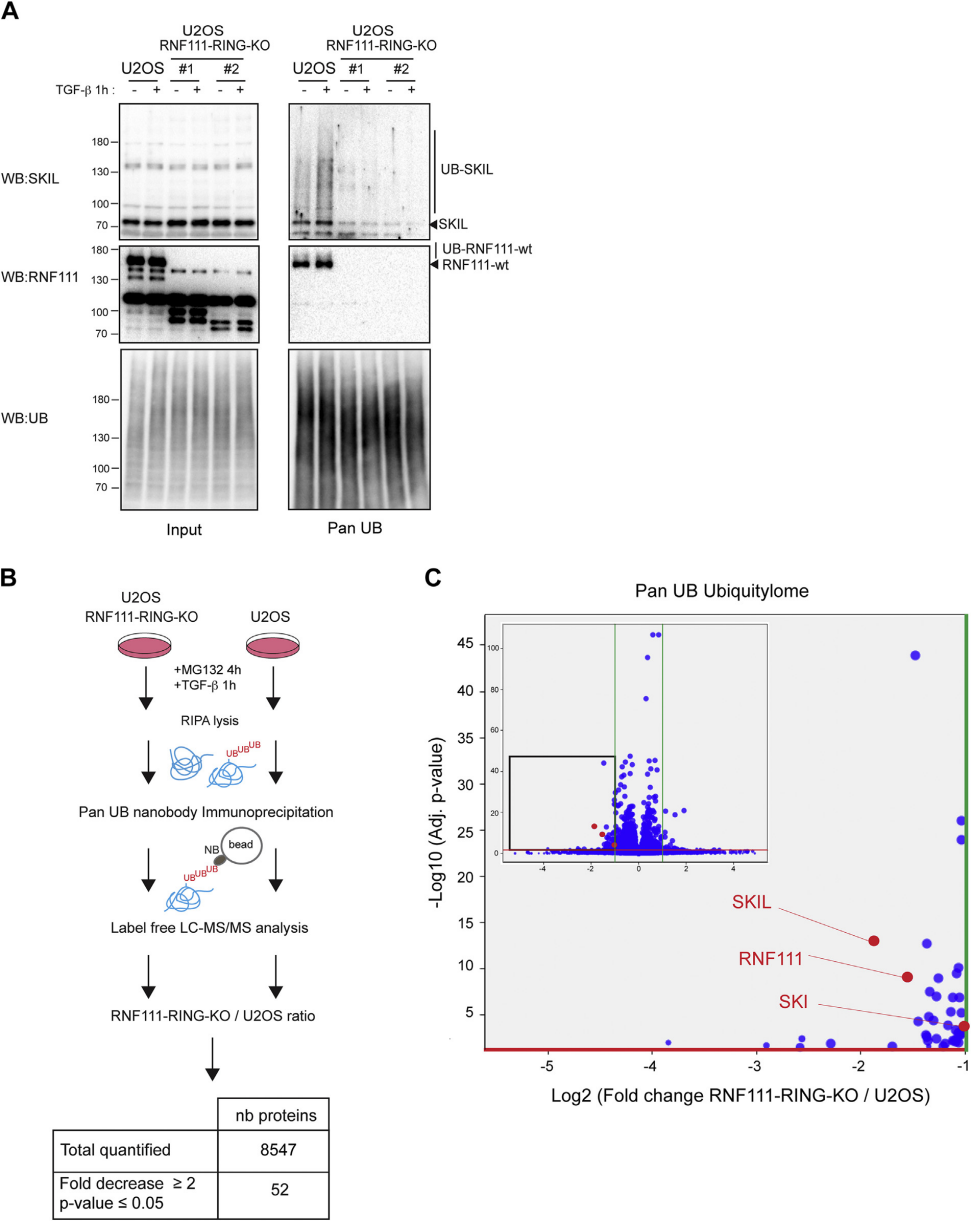
**Identification of RNF111-dependent substrates by pan UB ubiquitylome quantitative comparison.***A*, U2OS cells and RNF111-RING-KO clones #1 and #2 were treated with MG132 for 4 h before induction or not with TGF-β for 1 h. The whole cell lysates immunoprecipitated with the pan UB nanobody were analyzed by Western blotting (pan UB, *right panel*) along with whole cell lysates (input, *left panel*) using antibodies against RNF111, SKIL, and UB. *B*, schematic representation of the experimental design for RNF111-dependent ubiquitylome quantification with the pan UB approach using immunoprecipitation of ubiquitylated proteins with the pan UB nanobody. The table summarizes the results of the differential quantification for the RNF111-RING-KO/U2OS ratio. *C*, volcano plot representation of the differential analysis with the log_2_ fold change RNF111-RING-KO/U2OS *versus* the negative log_10_*p*-value. Each dot represents a protein. The *green* and *red lines* represent the cut-offs applied (respectively fold increase or decrease ≥2 and *p*-value ≤ 0.05). The plot is zoomed in the fold change decrease ≥ 2 and *p*-value ≤ 0.05 area. RNF111, ring finger protein 111; TGF-β, transforming growth factor-β; UB, ubiquitin.

Because the diGly approach led to the identification of numerous putative targets, we decided to refine our analysis by employing the pan UB nanobody resin to analyze the RNF111-dependent ubiquitylome. We first assessed whether immunoprecipitation with the pan UB nanobody enables the detection of endogenous SKIL ubiquitylation. Although TGF-β-induced SKIL degradation has been well documented (4, 5, 6, 29), no study has provided evidence of an increased ubiquitylation of SKIL upon TGF-β signaling, presumably because of the difficulty to detect endogenous ubiquitylation and because overexpression experiments temper this inducible effect. We found that immunoprecipitation with the pan UB nanobody enables the detection of increased endogenous SKIL ubiquitylation in the parental U2OS cells after 1 h TGF-β treatment in presence of proteasome inhibitor MG132, but not in the two RNF111-RING-KO clones despite the presence of equivalent amount of SKIL protein in the input of each condition (Fig. 4*A*). This result clearly demonstrates that TGF-β induces SKIL ubiquitylation, and that RNF111 E3 ubiquitin ligase activity is required in this process. Moreover, ubiquitylated RNF111 was also immunoprecipitated in the parental cell lines but not in the two RNF111-RING-KO clones that lack RNF111 auto-ubiquitylation ability. These results highlight that the pan UB nanobody constitutes a very efficient tool for endogenous purification of ubiquitylated proteins, and we then decided to carry out label-free proteomics on pan UB immunoprecipitated parental U2OS, RNF111-RING-KO clones #1 and #2 lysates. The easy workflow and the low amount of starting material of such an approach, compared with the diGly approach, allowed us to increase the number of biological replicates. We then compared four biological replicates of each RNF111-RING-KO clones #1 and #2 (RNF111-RING-KO clones n = 8) with 4 biological replicates of parental cells U2OS (U2OS n = 4) in the same conditions as the diGly experiments (4 h MG132, 1 h TGF-β). Among the 8547 quantified proteins, differential analysis enabled the detection of 52 proteins that display a statistically significant decrease of ubiquitylation in RNF111-RING-KO clones compared to parental U2OS cells (RNF111-RING-KO/U2OS fold decrease ≥ 2; *p*-value ≤ 0.05; Fig. 4, *B* and *C*, supplemental Table S4). Among these 52 putative RNF111 substrates, we identified SKI, SKIL, and RNF111, which confirms that the pan UB approach is very efficient for ubiquitylome analysis. Curiously, we also identified SMAD4 among the 52 hits but were not able to further validate that RNF111 indeed increases SMAD4 ubiquitylation (data not shown).

### Integrative Comparison of RNF111-Dependent Ubiquitylomes and Proteome

**Fig. 5:**
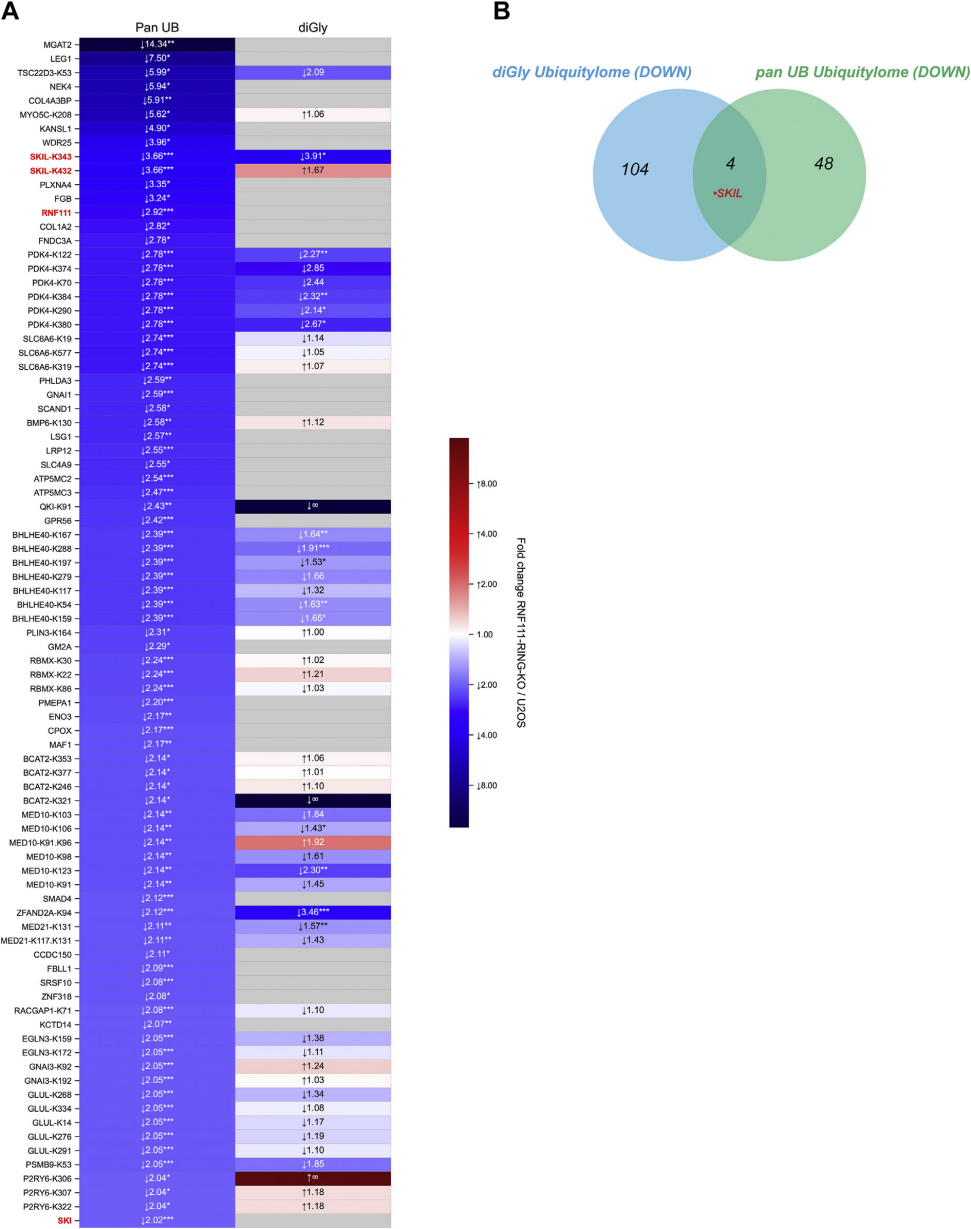
**Comparison of diGly and pan UB ubiquitylomes.***A*, the heatmap represents the ratios for the significant RNF111 substrate candidates in the pan UB ubiquitylome compared with the diGly ubiquitylome ratios. The ratios are annotated as fold increase or decrease, indicated by the *arrows*. *Asterisks* indicate their *p*-value: ∗*p* ≤ 0.05, ∗∗*p* ≤ 0.01, and ∗∗∗*p* ≤ 0.001. Note that we compared whole protein quantification in the pan UB case against ubiquitylation sites quantification for diGly (protein values may then be repeated when corresponding to multiple sites). *B*, Venn diagram comparison shows four common hits, including SKIL, between the pan UB and diGly ubiquitylomes significant RNF111 substrate candidates. diGly, diglycine; UB, ubiquitin.

To pinpoint the most robust RNF111 putative substrates, we next compared the 108 and 52 candidates obtained respectively with the diGly and pan UB ubiquitylome analyses (Fig. 5*A*). Strikingly, we identified only four common hits including SKIL and three other proteins PDK4, MED10, and ZFAND2A, (Fig. 5*B*). Because we were not able to validate these three candidates by Western-blot after pan UB immunoprecipitation neither at the endogenous level, nor after coexpression of an HA-tagged cDNA expression vector with Flag-RNF111 as for SKIL (data not shown), we concluded that SKIL constitutes the only common validated candidate for the diGly and pan UB ubiquitylome.

**Fig. 6:**
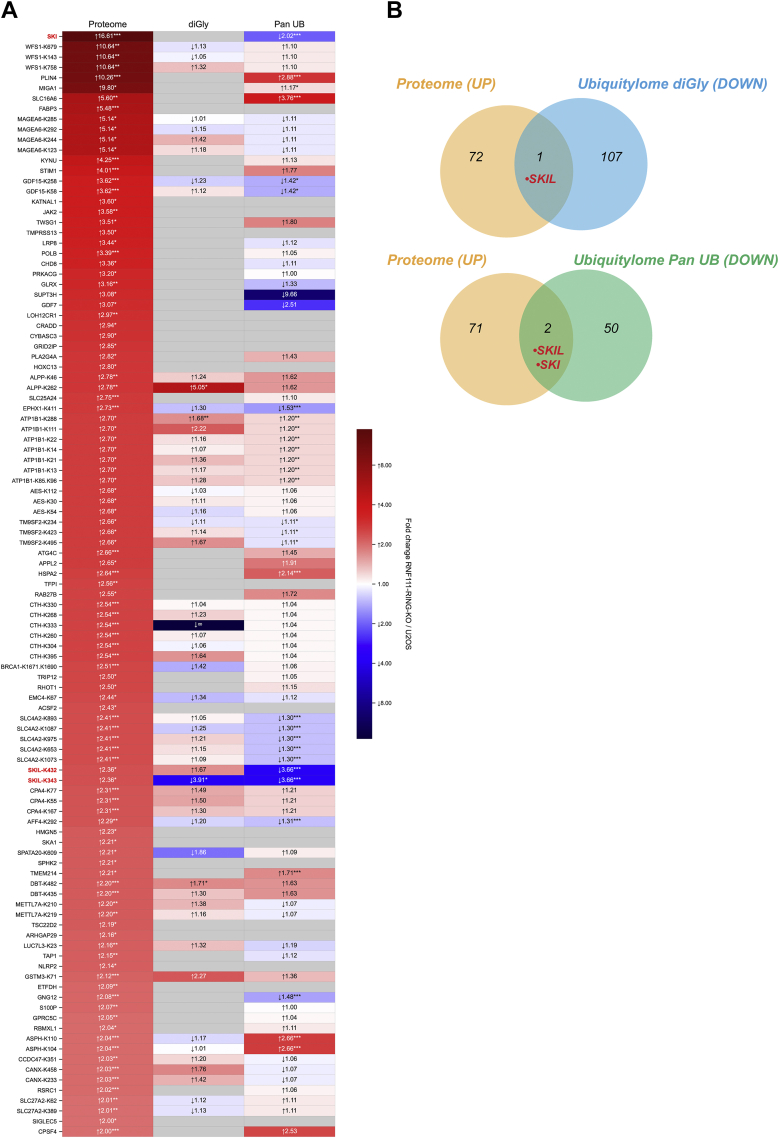
**Identification of RNF111 degradative substrates by comparison of RNF111-dependent proteome to diGly and pan UB ubiquitylomes.***A*, the heatmap represents the ratios for the significant RNF111 substrate candidates in the proteome, compared with pan UB and diGly ubiquitylomes ratios. The ratios are annotated as fold increase or decrease, indicated by the *arrows*. *Asterisks* indicate their *p*-value: ∗*p* ≤ 0.05, ∗∗*p* ≤ 0.01, and ∗∗∗*p* ≤ 0.001. Note that we compare whole protein quantification in the proteome and pan UB cases against ubiquitylation sites quantification for diGly (protein values may then be repeated when corresponding to multiple sites). *B*, Venn diagram shows SKI and SKIL as the only common hits between the proteome and pan UB or diGly ubiquitylomes significant candidates for RNF111 substrates. diGly, diglycine; RNF111, ring finger protein 111; UB, ubiquitin.

Finally, to identify substrates for RNF111 ubiquitin ligase function that are ubiquitylated and degraded by RNF111 in response to TGF-β, we performed an integrative comparison of the proteome with the two RNF111-dependent ubiquitylomes (Fig. 6*A*, supplemental Table S5). We identified SKIL as the only candidate with both decreased ubiquitylation and increased protein level in the absence of RNF111 ubiquitin ligase function when we compared the proteome with the diGly ubiquitylome, and SKI and SKIL as the only candidates when we compared the proteome with the pan UB ubiquitylome (Fig. 6*B*). Although it cannot be ruled out that other proteins than SKI and SKIL that were not detected can be ubiquitylated and degraded by RNF111, these results strongly argue that RNF111 specifically targets its substrates SKI and SKIL for degradative ubiquitylation in response to TGF-β.

## Discussion

### Lack of RNF111 E3 Ubiquitin Ligase Activity Affects Protein Expression at the Transcriptional Level

In the present work, we performed a comprehensive analysis of RNF111 substrates by using different quantitative proteomics approaches to compare parental U2OS cells with RNF111-RING-KO clones devoid of RNF111 E3 ubiquitin ligase activity. A proteome analysis enabled the identification of 73 proteins, including SKI and SKIL, with an increased level in the absence of RNF111 RING domain, suggesting that these hits could constitute new substrates of RNF111. However, further Western-blot and qPCR analysis of selected candidates indicates that RNF111 regulates these proteins at the transcriptional level independently of TGF-β, rather than affecting their protein stability. Although this could be an indirect effect, this finding raises the possibility that RNF111 could act as a transcriptional repressor. This would be in agreement with the study of Sun *et al*. (30), which performed transcriptomic analysis on MEF RNF111 −/− and identified a panel of genes repressed by RNF111, independently of TGF-β, that are also regulated by the Polycomb complex. Our study further indicates that RNF111 could regulate transcription in a RING dependent manner, suggesting that this effect might depend on a ubiquitylation event. However, we did not identify any components of the Polycomb complex in the ubiquitylomes or proteome that would be a target for RNF111 ubiquitylation and could explain this effect. Future investigations in this direction will be required to decipher the ability of RNF111 to directly repress transcription and to understand the relevance of its E3 ubiquitin ligase activity in this effect.

### Advantages and Limitations of the DiGly and Pan UB Ubiquitylome Analyses

Development of efficient approaches to enrich ubiquitylated proteins is a prerequisite to enable the identification of endogenous substrates of E3 ubiquitin ligases. Here, we have compared two methods of enrichment by performing immunoprecipitation with the diGly antibody and pan UB nanobody. DiGly remnant peptides enrichment followed by mass spectrometry analysis constitutes the most powerful method used to profile endogenous ubiquitylation and gives the major advantage to profile ubiquitylated lysines. However, because it involves detection of a single peptide for each ubiquitylation site, this method displays a low sensitivity that requires considerable amount of starting material and can be quite challenging to set up. In our study, we used a recently developed pan UB nanobody that empowers enrichment of ubiquitylated proteins at the endogenous level to perform ubiquitylome analysis. We found that this method is highly sensitive and much easier to implement than the diGly approach, and can therefore constitute an easy workflow alternative to investigate E3 ubiquitin ligases substrates profiling. Interestingly, the current development of nanobodies targeting the different polyubiquitin chain linkages will provide the opportunity in the future to investigate the identification of substrates for an E3 ubiquitin ligase according to the different ubiquitin linkages.

Comparison of the candidates obtained from our diGly and pan UB ubiquitylome analyses indicates that they poorly overlap, since only four common hits including SKIL have been identified and only SKIL could be validated. This suggests that ubiquitylome analyses might be significantly poised with false positive candidates due to biological noise. However, the discrepancy between the two ubiquitylomes approaches could also be explained by the difference in the two antibodies used for ubiquitin enrichment. Indeed, whereas the pan UB nanobody is specific of monoubiquitin and all polyubiquitin linkages, the K-ε-GG antibody used in the diGly approach is unable to distinguish between the attachment of ubiquitin and two other ubiquitin-like proteins NEDD8 and ISG15 that also leave a diGly remnant after trypsic digestion. Therefore, it is possible that some of the RNF111-dependent diGly peptides identified could correspond to a neddylation or ISGylation event. Indeed, it has been proposed that RNF111 could act as a NEDD8 E3 ligase (12, 31), and it will therefore be interesting in a future work to determine if RNF111 could neddylate some of the different candidates identified in the diGly ubiquitylome.

### SKI and SKIL are the Only Identified Degradative Substrates for RNF111-Ubiquitin Ligase Function in TGF-β Signaling

In the present work, we showed that comparison of the RNF111-dependent proteome and ubiquitylomes leads to the identification of SKI and SKIL as the only substrates ubiquitylated and degraded by RNF111 upon TGF-β signaling. Notably, we provide clear evidence for the first time that SKIL ubiquitylation is increased by TGF-β stimulation, and that RNF111 is absolutely required for this inducible effect. We are aware that our finding is not a proof that SKI and SKIL constitute the only endogenous substrates of RNF111, because we cannot rule out that some other substrates have not been detected in our screen. However, the important representativeness of our study with more than 7700 proteins identified in the proteome and the pan UB ubiquitylome and 12,000 ubiquitylation sites identified in the diGly ubiquitylome indicates that despite the paradigm of the ability for an E3 ubiquitin ligase to target many different substrates, RNF111 ubiquitin ligase function seems to display a very stringent specificity for its degradative substrates SKI and SKIL upon TGF-β pathway activation. On the other hand, the comparison of the RNF111-dependent ubiquitylomes to the proteome indicates that most of the putative ubiquitylated substrates of RNF111 are not associated with a significant increase in protein level, which could suggest that such proteins are non degradative ubiquitylation substrates for RNF111. The fact that RNF111 has been reported to trigger K27 and K63 non degradative ubiquitylation corroborates this possibility (11, 27). Further investigation will be required for the validation and significance of these potential non degradative ubiquitylation events. However, our comparison of the pan UB and diGly ubiquitylomes which identifies SKIL as the only common validated substrate further supports the idea that SKIL constitutes the major ubiquitylated substrate for RNF111. Importantly, our study has been performed in the presence of an active TGF-β signaling pathway, and it is likely that RNF111 affects ubiquitylation only in a stimuli-dependent manner. To depict the ubiquitylation network of RNF111, it would be interesting to employ the same approach to identify targets of RNF111 in response to other stimuli where RNF111 has also been involved, such as response to arsenite treatment (10), UV (11) or IR irradiation (12).

### Lysine K343 of SKIL Is a Ubiquitylation Target for RNF111

The advantage of the diGly approach is that it enables detection of the lysines that are ubiquitylated on the substrate. We found that ubiquitylation of lysine 343 of SKIL is dependent of RNF111. However, the mutation of lysine 343 together with the adjacent lysine 342 did not result in attenuated SKIL ubiquitylation. A previous study on overexpressed SKIL lysine mutants has shown that SKIL degradation in response to TGF-β depends on lysine 440, 446, and 449, but no ubiquitylation experiments have confirmed this observation (29). It is, however, possible that RNF111 ubiquitylates multiple lysines on SKIL including lysine 343, and other lysines such as lysine 440, 446, and 449 that were not detected in the ubiquitylome. A more targeted approach by SKI and SKIL immunoprecipitation followed by diGly enrichment would enable to map precisely all the lysines ubiquitylated by RNF111 on SKI and SKIL. Alternatively, it is possible that lysine 343 is indeed the only endogenous ubiquitylation target for RNF111, and that overexpression of SKIL and RNF111 triggers ubiquitylation toward other lysines. Intriguingly, the lysine 343 is located in the SAND domain of SKIL known to interact with SMAD4 (28) and RNF111 (5). It has been shown recently that besides its interaction with phospho-SMAD2/3 (4), interaction of SKIL with SMAD4 is also absolutely required for SKIL degradation (32). Altogether, these findings point out that RNF111 ubiquitylation of SKIL might occur at the interface of a SKIL–SMAD4–phospho-SMAD2/3 heteromeric complex, and future investigation in this direction would be critical to unravel the molecular mechanism of RNF111-dependent SKIL degradation in response to TGF-β.

In conclusion, by showing that SKI and SKIL constitute the main degradative substrates for RNF111 E3 ubiquitin ligase function in TGF-β signaling, our integrative proteomics analysis indicates that RNF111 displays a strong specificity toward its substrates. This finding further suggests that drugs targeting RNF111 E3 ubiquitin ligase function would specifically enable the inactivation of TGF-β signaling by preventing SKI and SKIL degradation.

## Data availability

The mass spectrometry proteomics data, including peptide quantification, have been deposited to the ProteomeXchange Consortium *via* the PRIDE (33) partner repository identified with the dataset identifier PXD025890.

## Supplemental data

This article contains supplemental data.

## Conflict of interest

The authors declare no competing interests.

## Figures and Tables

**Supplemental Figure S1:**